# Expression of lipoma preferred partner in mammary and extramammary Paget disease

**DOI:** 10.1097/MD.0000000000023443

**Published:** 2020-12-18

**Authors:** Ye Liu, Yangbin Wang, Ruiqun Qi, Xiaoyun Mao, Feng Jin

**Affiliations:** a Department of Breast Surgery, The First Affiliated Hospital of China Medical University; b Department of Dermatology, The First Hospital of China Medical University, Heping District, Shenyang, Liaoning Province, P.R. China.

**Keywords:** cell motility, immunohistochemistry, lipoma preferred partner, Paget disease

## Abstract

**Backgound:**

This study aims to identify the expression of lipoma preferred partner (LPP) in Paget disease (PD) and to further understand the pathogenesis of PD.

**Methods:**

Tissue microarray was used to evaluate the expression of LPP by immunohistochemistry in 40 PD patients. The results of LPP expression were combined with clinical and histopathological characteristics. Patient files were analyzed retrospectively.

**Results:**

Twenty-one cases were mammary Paget disease (MPD) and 19 extramammary Paget disease (EMPD) involving the vulva, scrotum, and penis. LPP was expressed in PD and this expression was significantly greater in MPD versus EMPD (*P* = .031). The expression of LPP in MPD was significantly related with age (*P* = .009) and expression of Ki-67 (*P* = .011). No statistically significant differences were observed in LPP expression as related to sex, body location, and time of PD diagnosis.

**Conclusions:**

While LPP is expressed in both MPD and EMPD, the intensity of this expression is greater in MPD. LPP expression is positively correlated with Ki-67 and is more prevalent in middle-aged versus senior MPD patients. Further research is needed to determine its potential role in tumorigenesis and distribution.

## 1 Introduction

Paget disease (PD), which is a malignant tumor of the skin, is similar to eczema and therefore also referred to as eczema-like cancer. PD is often misdiagnosed which leads to delays in treatment and affects its prognosis. Cutaneous PD can be classified as being either “mammary” or “extramammary.” The basis for this classification is attributable to 2 historical events: Dr James Paget first described mammary Paget disease (MPD) in 1874.^[1]^ And 25 years later, Radcliffe Crocker described Extramammary Paget disease (EMPD) as an independent type.^[2]^ As a number of people consider these diseases as indistinguishable histopathologically, this dichotomous classification remains intact. MPD accounts for 1% to 3% of primary breast tumors and is often accompanied with ductal carcinoma in situ or invasive ductal carcinoma. It frequently originates in the nipple/areola complex region and can then spread to the surrounding skin.^[3]^ In 2017, Zhao et al^[4]^ reported that 2962 of 3431 MPD patients presented with ductal carcinoma in situ or invasive ductal cancer (the data are from SEER). Although EMPD can occur in any part of the skin or mucous membrane, it is most commonly observed in the anal-genital area,^[5]^ accounting for 1% of primary vulvar tumors.^[6]^ EMPD can be divided into primary or secondary EMPD, with secondary EMPD usually involving skin metastasis of other malignant tumors, such as rectal, bladder, endometrial, and gastric cancers.^[7]^

Although MPD and EMPD are both considered as PD, there remains the question of whether they are one, in the same, disease. Identification of the origin/differentiation of Paget cells (PCs) may provide one possibility to address this issue. EMPD shares some targetable biomarkers with its mammary counterpart,^[8]^ however the histogenesis of PD is controversial. Within our clinic we have been distinguishing MPD from EMPD as based upon the location of the disease, but the validity of this criterion is questionable. As MPD and EMPD differ in terms of treatment and prognosis, it is imperative to correctly distinguish between the 2. The most accepted current belief is that in MPD, PCs migrate from the ductal breast cancer cells to the nipple through the basement membrane,^[9]^ while EMPD originates from the apocrine glands.^[10]^ MPD is determined using a combination of clinical, imaging and histopathological information, and treatment depends on whether it is accompanied by breast cancer, as well as whether lymph node metastasis is present. Regardless of whether other signs of malignancy are observed, mastectomy is considered the treatment of choice for MPD.^[2]^ With regard to treatment of EMPD, a detailed inspection must initially be performed to determine the presence of related malignancies, especially in cases where perianal lesions are present. Surgical resection and microsurgery represent the best treatment options for EMPD, which must be closely monitored for an indefinite period as it often recurs.^[2]^

Lipoma preferred partner (LPP) is a member of the zyxin family of proteins, whose main function is to regulate cytoskeletal organization, cell movement, and mechanical sensitivity.^[11]^ Recent findings have revealed that LPP plays an important role in tumor cell migration, invasion, and metastasis.^[11]^ LPP has the ability to enter the nucleus and localize to focal adhesion, and has been reported to promote the formation of invadopodia.^[11]^

An estimated 90% of cancer deaths result from metastatic diseases.^[12]^ Invasive cancer cells acquire their capacity for invasion by forming invadopodia.^[12]^ Invadopodia enables cancer cells to escape from the primary tumor, break through blood vessel barriers, and implant into distant tissue.^[13,14]^ With the development of in vivo cell imaging technology, we have recently been able to observe this process in vivo^[15–17]^ and found that in the early stages of the metastasis, cancer cells combine with invadopodia and destroy endothelial cells. Perhaps not too surprisingly, invadopodia has been found in many invasive cancer cell lines, such as breast, head, and neck and prostate cancers, as well as in fibrosarcoma and melanoma.^[13]^ Related to these findings on invadopodia, is the report that LPP has been shown to be a key mediator in inducing the migration and invasion of breast cancer cells.^[18]^ However, there are currently no reports on LPP expression in PD. Therefore, the purpose of this study was to determine whether LLP expression is present in PD and assess its potential role in this condition.

## 2 Materials and methods

### 2.1 Study group

In this report a retrospective review of Chinese PD patients’ records was performed. The clinical data were retrieved from the electronic case system and included age, sex, lesion localization, number of PCs per unit of epidermis area, length of epidermal process, acanthosis thickness, infiltration depth of inflammatory cells in dermis, and immunohistochemical results of ER, PR, Ki-67, C-erbB-2 in MPD were recorded.

### 2.2 Collection of PD samples

PD cases (N = 40) consisting of 21 MPD and 19 EMPD patients treated at the First Affiliated Hospital of China Medical University over the period from January 2010 to January 2015 were analyzed. All cases were confirmed by a pathologist. The 21 MPD samples were all female patients, with 11 showing MPD in the left breast and 10 in the right breast. Of the 19 EMPD samples, 13 were from men (10 scrotal, 2 penile, and 1 axillary) and 6 women (5 in the vulva and 1 axillary).

### 2.3 Tissue microarrays (TMA)

Wax blocks and sections of the 40 tissue samples were collected from the First Affiliated Hospital of China Medical University and were assessed microscopically. A 1 to 2 mm point within each section was selected and a semi-automatic tissue drill was used to isolate a tissue cylinder (1–2 mm in diameter) around or in the center of the donor block, where a large number of tumor cells and almost no necrosis was located. These isolated samples were then inserted into 2 new wax blocks for TMA. TMA at 3 μm thickness were sliced adjacently using an automatic paraffin section system. Immunohistochemistry was performed after sectioning.

### 2.4 Immunostaining

The TMA sections were deparaffinization and rehydrated using xylene and graded alcohol. The sections were then blocked with 2% bovine serum albumin, incubated with LPP-specific primary antibodies for 2 hours, followed by incubation with biotinylated secondary antibodies for 1.5 hours and then HRP-bound streptavidin. Subsequently, diaminobenzidine (DAB) was added dropwise over a 3 to 5 minute period and sections were then counterstained with hematoxylin. LPP antibody was a Rabbit anti human polyclonal antibody purchased from USBiological Life Sciences Company (Catalog No: NP_001161143) and was used at a dilution of 1:100.

### 2.5 Sample assessment

The immunohistochemical results of the 2 tissue chips were scanned using electronic scanning and were then observed and analyzed using CaseViewer. LPP positive staining was cytoplasmic. Following the procedure of Campo et al,^[19]^ the results were assessed by combining the dyeing strength and dyeing percent. The dyeing strength was assigned the following values: 0—basic non staining, 1—weak staining, 2—medium staining, and 3—strong staining. The dyeing percent was assigned the following values: 0 points—0% of the total cells were stained, 1 point—6% to 25% staining, 2 points—26% to 50% staining, and 3 points—>50%. Results were presented as based upon the sum of staining intensity and percent: 0—negative (–), 1 to 2—weak positive (+), 3 to 4—medium positive (+ +), and ≥5—strong positive (+ ++).

In addition to evaluating staining intensity, we also determined the number of PCs per unit area, the acanthosis thickness, the length of epidermal processes, and the infiltration depth of inflammatory cells in dermis as assessed using CaseViewer. The invasion of PCs in the dermis, spinous layer loosening, and involvement of appendages in each sample were also determined. Calculation of the number of PCs per unit area consisted of randomly selecting 3 regions at the 2 ends and the center of the epidermis of each sample. The number of PCs in each region were counted and divided by the corresponding region area with the final calculation derived from the average of the 3 regions.

### 2.6 Statistical analysis

Analysis was performed using the IBM SPSS Statistics 25 program (SPSS Inc., Chicago, IL). Statistical tests included Chi-squared-tests, independent sample *t* tests, one-way analysis of variance, and the rank sum test to evaluate the relationship between the immunostaining and clinicopathological parameters. A *P* < .05 was required for results to be considered as statistically significant.

## 3 Results

### 3.1 Patient characteristics

Demographic and clinicopathological characteristics of the patients are summarized in Table 1. The average age of the MPD patients was 61.0 years and EMPD patients was 63.2 years. These differences in age between samples from MPD and EMPD patients were not statistically significant.

**Table 1:** Summary of demographic and clinical-pathologic findings in patients with Paget disease (PD).

EMPD (19)		MPD (21)	
Characteristic	Value	Characteristic	Value
Age, y		Age, y	
Mean, median	63.2,61.5	Mean, median	61.0,63.0
Min, max	41.0,89.0	Min, max	38,80
Sex, n		Anatomic site, n	
Male	13	Left breast	11
Female	6	Right breast	10
Anatomic site, n		Number of PCs per unit	
Vulva	5	Epidermis area, n/mm^2^	
Scrotum	10	Mean, median	550, 500
Penis	2	Min, max	110, 2100
Axilla	2	Depth of inflammatory cells in	
Number of PCs per unit		Dermis, μm	
Epidermis area, n/mm^2^		Mean, median	293, 260
Mean, median	250, 220	Min, max	0, 932
Min, max	60, 920	Acanthosis thickness, μm	
Depth of inflammatory cells in		Mean, median	219, 190
Dermis, μm		Min, max	55, 560
Mean, median	351, 359	Length of epidermal process, μm	
Min, max	80, 621	Mean, median	168, 147
Acanthosis thickness, μm		Min, max	40, 359
Mean, median	316, 265	ER status, n	
Min, max	140, 720	Positive	6
Length of epidermal process, μm		Negative	12
Mean, median	221, 185	Not know	3
Min, max	70, 440	PR status, n	
		Positive	5
		Negative	11
		Not know	5
		Ki-67 status, n	
		Positive <15%	4
		Positive >15%	13
		Not know	4
		C-erbB-2 status, n	
		2+	6
		3+	9
		Not know	6
		Ulceration of MPD	
		Yes	15
		No	6
		Nipple discharge of MPD	
		Yes	14
		No	7

### 3.2 Pathological features

The PCs in all cases of MPD and EMPD showed atypical round or oval nuclei, were rich in basophils, with a hermaphroditic or transparent cytoplasm. LPP was mainly expressed in the cytoplasm of PCs with the color of brown and yellow. A similar morphological appearance of the PCs was observed in MPD and EMPD samples. PCs formed single cell layers or clusters, which were located in all layers of the epidermis. The acanthosis thickness was 218.9 μm in MPD and 315.9 μm in EMPD samples (*P* = .043). There was no significant differences in the number of PCs per unit of epidermal area (*P* = .211), length of epidermal processes (*P* = .091), or infiltration depths of inflammatory cells in the dermis (*P* = .715) between MPD and EMPD samples.

### 3.3 Expression of LPP was greater in MPD versus EMPD samples

**Figure 1:**
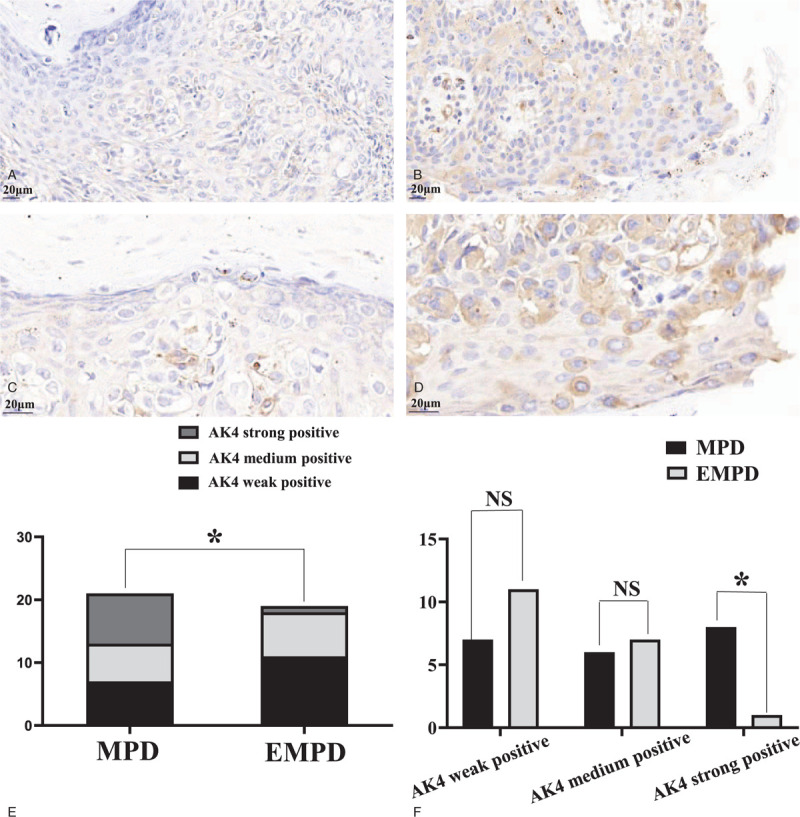
Representative images of immunohistochemical staining from MPD and EMPD samples. LPP expression in PCs (A) low expression in EMPD, ×400; (B) high expression in MPD, ×400; (C) low expression in EMPD, ×728; (D) high expression in MPD, ×728; (E) there was a statistically significant difference in LPP expression between MPD and EMPD samples (rank sum test, ∗ present *P* ≤ .05); (F) high expression of LPP in the MPD versus low expression of LPP in the EMPD group (chi-squared-tests, ∗ present *P* ≤ .05, NS present not significant). EMPD = extramammary Paget disease; LPP = lipoma preferred partner; MPD = mammary Paget disease; PC = Paget cells.

Immunohistochemical staining revealed that LPP was expressed in the cytoplasm in both MPD and EMPD samples (Fig. 1), with all cases of MPD and EMPD showing positive staining for LPP. In MPD samples, the numbers of strong, medium, and weak positive staining were 8, 6, and 7, respectively, while in EMPD these numbers were 1, 7, and 11. These results indicated a statistically significant difference (*P* = .031) with the overall expression of LPP intensity being greater in the samples from the MPD versus EMPD group.

### 3.4 Expression of LPP was greater in middle-aged group versus senior group in MPD

**Figure 2:**
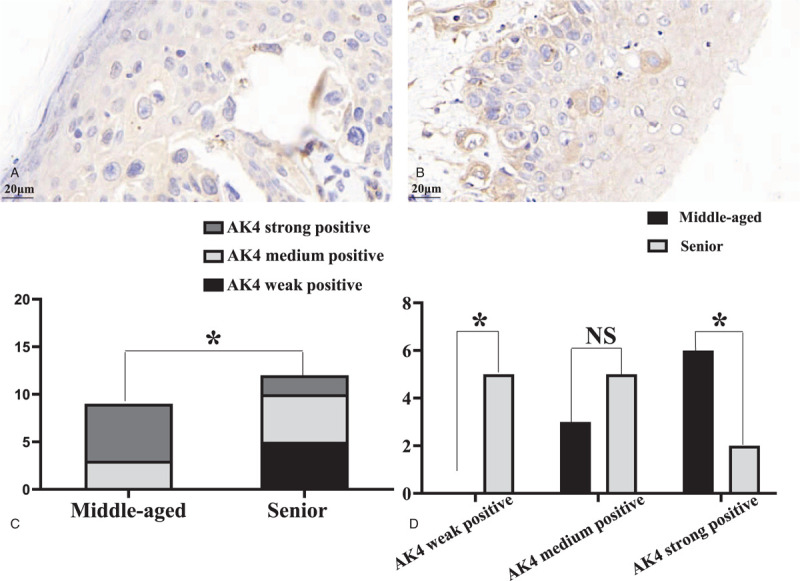
Expression of LPP in MPD samples from 2 different age groups. Expression of LPP in PCs of MPD samples in (A) senior group, ×728; (B) middle-aged group, ×728; (C) there was a statistically significant difference in LPP expression between the middle-aged and senior group (rank sum test, ∗ present *P* ≤ .05); (D) LPP high expression in middle-aged group versus LPP low expression in senior group (chi-squared-tests, ∗ present *P* ≤ .05, NS present not significant). LPP = lipoma preferred partner; MPD = mammary Paget disease; PC = Paget cells.

In samples from MPD patients, 9/21 were from patients under 60 years of age (middle-aged group) and 12/21 from patients >60 years old (senior group), Within the middle-aged group, the number of samples showing strong, medium, and weak LPP staining were 6, 3, and 0, respectively, while these numbers within the senior group were 2, 5, and 5 (Fig. 2). These results indicated a statistically significant difference (*P* = .009) with the overall expression of LPP intensity being greater in samples from the middle-aged versus senior MPD group.

### 3.5 Expression of LPP was greater in Ki-67 >15% group versus Ki-67 <15% in MPD

**Figure 3:**
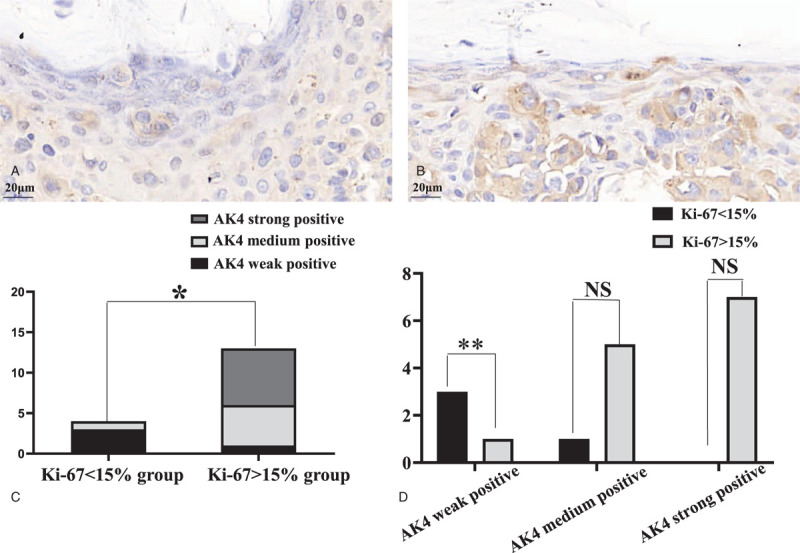
The expression of LPP in MPD samples within 2 different Ki-67 status groups. Expression of LPP in PCs of MPD samples in (A) Ki-67 <15% group, ×728; (B) Ki-67 >15% group, ×728; (C) there was a statistically significant difference in LPP expression between the Ki-67 <15% and Ki-67 >15% group (rank sum test, ∗ present *P* ≤ .05); (D) LPP high expression in Ki-67 >15% group versus LPP low expression in Ki-67 <15% group (chi-squared-tests, ∗∗ present *P* ≤ .01, NS present not significant). LPP = lipoma preferred partner; MPD = mammary Paget disease; PC = Paget cells.

In MPD samples, there were 13 cases in Ki-67> 15% group and 4 cases in Ki-67 <15% group. In the Ki-67 >15% group, the numbers of strong, medium, and weak staining of LPP were 7, 5, and 1, respectively, while in the Ki-67 <15% group these numbers were 0, 1, and 3 (Fig. 3). These results indicated a statistically significant difference (*P* = .011) with overall expression of LPP intensity being greater in the Ki-67 >15% versus Ki-67 <15% group.

### 3.6 Association of LPP expression with expressions of EP, PR, and C-erbB-2 in MPD

No statistically significant correlations between LPP expression and that of EP, PR, and C-erbB-2 expressions were obtained in these MPD samples (Table 2).

**Table 2:** Clinicopathological characteristics in relation to LPP expression status.

	Entire group	LPP			
Variable	(n = 26)	Weak	Medium	Strong	*P* value
Ulceration, n					.48
Yes	15	4	6	5	
No	6	1	2	3	
Nipple discharge					.179
Yes	14	3	5	6	
No	7	3	4	0	
ER status					.638
Positive	6	1	2	3	
Negative	13	3	5	5	
Not know	5				
PR status					.855
Positive	5	1	2	2	
Negative	11	2	4	5	
Not know	5				
C-erbB-2 status, n					.611
2+	6	1	3	2	
3+	9	2	2	5	
Not know	6				

## 4 Discussion

A considerable number of studies have been performed in which samples from PD patients have been compared with those of normal skin in an attempt to identify biomarkers that may be specific for PD. However, few studies exist which have examined potential biomarker differences between MPD and EMPD. In this report, we focused on the identification of potential marker differences between MPD and EMPD, and therefore did not include samples from normal skin tissue.

Results from numerous cell and transgenic mouse model studies indicate that LPP plays a role in the invasion and metastasis of some cancers, such as breast,^[20]^ ovarian,^[21]^ endometrial,^[22]^ and lung cancers.^[23]^ However, to the best of our knowledge, no studies have directed toward examining the expression of LPP in PD. In this study, we determined LPP expression in samples from MPD and EMPD patients and related these expression profiles with clinicopathological features of these conditions. When comparing the MPD group with that of the EMPD group, we found that the expression of LPP and the number of PCs per unit epidermis area were significantly higher in the MPD group, while acanthosis thickness and length of epidermal processes were significantly higher in the EMPD group. Within the MPD group, we found that the expression intensity of LPP in the middle-aged group was significantly increased as compared with that in the senior group, and Ki-67 expression was positively related to LPP in MPD. Such findings provide new insights the origin and prognosis of PD and the capacity to discriminate between MPD and EMPD.

MPD and EMPD represent rare intraepithelial neoplasias with similar clinical features that may resemble inflammation and infectious skin disease.^[24]^ Over 90% of MPD cases originate from in situ or infiltrative lower breast cancer,^[3]^ while only 5% to 30% of EMPD cases are associated with lower breast cancer.^[25]^ Currently, the underlying mechanisms responsible for MPD remain controversial. Two recognized mechanisms, and a third hypothesis have been proposed.^[2]^ The first is the epidermotropic theory which postulates that PCs may migrate from intraductal breast cancer cells to the papilla through the basement membrane.^[9]^ This theory is supported by data showing the similarity between PCs’ immunohistochemical staining and the underlying cancer.^[26]^ The second theory postulates that PCs are malignantly transformed keratinocytes, which are in situ carcinomas regardless of the underlying ductal cancer.^[27]^ Support for this theory has been provided from findings of ultrastructural studies, which showed that microvilli and desmosomes were found between PCs and local keratinocytes.^[28]^ The third theory represents a combination of the first 2, and proposes that PCs can be generated in both of the 2 ways described above, depending on local conditions.^[5]^ EMPD is considered to be an adenocarcinoma that originates in skin or skin appendages within apocrine glands.^[10]^ In the past, the identification of PD and eczema have been considered like that of other skin malignancies, such as Bowen disease and primary melanoma. As the early manifestation of PD involves squamous erythema, it is easy to be misdiagnosed as eczema. A small number of PD cases show significant amounts of abnormal cells, called anaplastic PD. These abnormal cases of PD can be difficult to distinguish from Bowen disease or melanoma, when evaluated using conventional pathological examinations. This is especially problematic when the PD is accompanied by pigmentation, which is then more likely to be confused with melanoma. Therefore, it is necessary to simultaneously combine immunohistochemical examinations with routine pathological examinations. Bowen disease is generally negative for CK7 and CEA and can occur in any part of the body. Its presence in the nipple and scrotum should alert the clinician as to the possibility of MPD. Differentiation of PD from melanoma in situ mainly depends on immunohistochemistry, where S-100, HMB45, and Melan-A positive proteins can be identified.

MPD and EMPD do differ in some aspects. Most MPD cases are accompanied by latent breast cancer (either infiltrating or in situ),^[3]^ while only a few cases of EMPD involve direct extensions of latent adenocarcinoma. The prognosis and treatment of the 2 also differs. MPD, and secondary EMPD, are often accompanied by or associated with other tumors. Therefore, methods of treatment include resection of the tumor and free surgical borders. In contrast, primary EMPD does not have underlying tumors and can be treated by chemotherapy or surgery to remove affected skin with clear edges.^[29]^ However, MPD and EMPD appear identical histologically, and cannot be distinguished when viewed under a microscope. For example, as based upon results of a hematoxylin-eosin study, it was reported that MPD and EMPD failed to differ significantly with regard to several histopathology criteria.^[30]^ Much effort has been directed toward identifying histopathological differences between MPD and EMPD. The most common markers associated these conditions include estrogen receptor (ER), progesterone receptor (PR), CEA, and C-erbB-2,^[5]^ however none of these markers alone proved irrefutably reliable, as these can be found in varying degrees within both types of PD. Some potentially promising results were in the findings that MPD and EMPD expressed different mucin antibodies.^[31]^ EMPD expressed MUC5AC and could not express MUC2, while MPD did not express MUC5AC. However, during MUC5AC immunostaining, some studies found that MUC5AC only labeled a small number of mainly scattered PCs.

An important question regarding a distinction between MPD and EMPD is whether an “EMPD-type” of Paget disease would be located in the breast. In 2004, Ohnishi et al^[32]^ reported on a case of a 91-year-old woman with areola, axillary, and genital lesions. These 3 sources of lesions showed similar immunophenotypes, including MUC5AC immunoexpression. Based upon these findings, the authors suggested that some cases of MPD may actually be EMPD cases.^[32]^ This rare case and innovative hypothesis have largely been ignored in the literature. However, in 2018, Fernandez-Flores et al^[29]^ reported 5 cases of an “EMPD-type” Paget disease in the breast. Therefore, we can indeed acknowledge the presence of EMPD in the breast. Such cases show a protracted development and can benefit from non-radiative treatments. The overall survival rate of EMPD is similar to that of the general population.^[33]^ Therefore, the findings of an “EMPD-type” Paget disease in the breast highlight the significance of correctly discriminating between MPD and EMPD with regard to subsequent treatment and prognosis. Our current findings demonstrate that the expression of LPP in the MPD group was higher than that in the EMPD group. Accordingly, LPP levels may serve as a potential reference for the identification of MPD versus EMPD. These differences in the expression of LPP in MPD versus EMPD may indicate that they originate from different tissues, that is they represent 2 distinct diseases, but additional evidence from further studies will be required to confirm this eventuality.

Directed cell migration plays an important role in the normal development and function of multicellular organisms and also participates in cancer progression by disseminating the distribution of these tumor cells. The interaction of cancer cells with the surrounding extracellular matrix (ECM) affects the mechanisms that promote cell migration and invasion. In cancer, increased cell migration is at the core of cancer cell metastasis. With their capacity to migrate through the interstitial ECM around the primary tumor, cancer cells can enter the vascular system, eventually reaching distant organs and tissues.

Cancer cell metastasis is the main cause of death in cancer patients.^[12]^ Cancer cells need to cross several physical barriers to escape from the primary tumor and distribute into the bloodstream and other tissues.^[12]^ Invasive cancer cells cross these barriers by forming invadopodia.^[34]^ Invadopodia are present on the ventral side of invasive cancer cells and are rich in actin complexes, including wasps, Arp2/3, cortical actin, tks4/5, and c-Src.^[35–40]^ In addition, they can locally degrade the ECM by activating MMP2, MMP9, MT1-MMP, ADAM12, adam15, and ADAM19 proteases.^[41]^ Invadopodia protrude from the ventral side of the plasma membrane of cancer cells and directly contact the ECM.^[34]^ The cell surface components of invadopodia are mainly composed of integrins and proteases whose functions are consistent with the presumed role of invadopodia in malignant tumor cells, that is, to degrade the ECM and provide traction for malignant tumor cells.^[34]^ The internal components of invadopodia have 3 main functions, signal transduction, cytoskeletal assembly, and membrane transport.^[42]^ The combination of these cellular activities enables invadopodia to function as an ideal invasive machine that responds to external signals, generates forces to alter cell shape, and concentrate molecules at predetermined locations to further degrade and transport the ECM.^[34]^

The protein-containing LIM domain is a major regulator of various cellular processes and plays a key role in regulating the actin cytoskeleton. The LPP gene for lipomas is located on chromosomes 3q27-q28 and is characterized by the continuous rearrangement of chromosomal fragments of high mobility group gene alleles at 12q15.^[43]^ LPP is localized to focal adhesions (FAs),^[44]^ and its N-terminal domain, which interacts with α-actin^[45]^ and Ena/VASP,^[46]^ is related to the actin cytoskeleton. It has long been believed that LPP promotes the migration of mesenchymal/fibroblasts. Recent evidence has indicated that LPP has become an important inducer of tumor cell migration, invasion, and metastasis. Although LPP is unnecessary for tumor growth, it is essential for tumor invadopodia formation, extravasation of breast cancer cells, and establishing lung metastases.^[47]^ Results from previous studies have shown that LPP can contribute to the metastasis and invasion of cancer cells, such as breast,^[20]^ ovarian,^[21]^ endometrial,^[22]^ and lung cancers.^[23]^ To accomplish this function, LPP initially localizes to focal adhesions through its LIM1 domain after TGFB stimulation and then recruits a-actin to stimulate breast cancer cell migration and invasion.^[20]^ Src tyrosine kinase mediates TGFB induced tyrosine phosphorylation of LPP, which is required for the formation of invadopodia and ECM degradation.^[47]^ In ovarian cancer, cancer-associated fibroblasts (CAFs) upregulate LPP in microvascular endothelial cells (MECs).^[21]^ The resultant increase in LPP expression promotes the formation of focal adhesion complexes, increases cell traction in endothelial cells, and increases leakage in the endothelial cell monolayer, which promotes the mobility and permeability of endothelial cells.^[21]^ Epithelial-mesenchymal transition (EMT) is considered to be a key process for tumor invasion^[22]^ and results from molecular studies have confirmed that ETV5 has direct effect on EMT.^[22]^ Colas et al^[22]^ reported that Etv5 and LPP jointly receive extracellular signals to promote the occurrence of EMT in endometrial cancer.

Our current results show that LPP was expressed in both MPD and EMPD, but the expression intensity within MPD samples was greater than that of EMPD. Most of the MPD samples were found to be strongly positive, while most of the EMPD samples were weakly positive for LPP. We speculate that this disparity may be related to differences in their origins. At present, most investigators believe that MPD originates from potential breast cancer and that PCs in MPD originate from breast cancer intraductal cancer cells migrating to the nipple through the basement membrane. However, it is not known whether this migration of breast neoplastic cells to the nipple involves a passive, active, or combination of transport processes. Schelfhout et al^[48]^ reported that papillary keratinocytes can release a motility factor, hergulin-a, which can act on HER3 or HER4 or the HER2/neu receptor expressed by PCs to induce chemotaxis of PCs and subsequent distribution into the papillary epidermis. This process then plays a key role in the pathogenesis of MPD. Obviously, this can be seen as a passive metastasis process of breast neoplastic cells to the papillary epidermis under the action of motility factors. We speculate that this may be a mechanism for active transport of breast cancer cells to the papillary epidermis. Our current results offer some new perspectives on this issue. As a promoter of mesenchymal/fibroblast migration, LPP is involved in cancer cell migration and invasion in a variety of cancers. In breast cancer, under the stimulation of TGFB, LPP localizes to focal adhesion sites through the LIM1 domain and recruits a-actin to stimulate breast cancer cell migration and invasion, such as metastasis to the lungs. Under the action of LPP, breast cancer cells may also transfer to the nipple epidermis and these high expression levels of LPP in MPD PCs may be due to its internal breast cancer. In this way, these cells could either originate from the cancer cells in the duct or distribute to the skin via the infiltrating cancer cells. As we have only observed typical PCs in the epidermis, we speculate that the former possibility is more likely.

Ki-67, a proliferating cell-related antigen, was discovered in 1983. Ki-67 belongs to the class of non-histones and is currently recognized as marker of proliferation. Its function is closely related to mitosis and it is an indispensable component for cell proliferation.^[49]^ As one of the most widely used cell proliferation markers, Ki-67, can reflect the degree of malignant cell proliferation and is closely related to the progress, metastasis, and prognosis of various malignant tumors.^[50]^ When tracing the display of Ki-67 throughout the cell cycle, there is no expression in GO, an initial appearance in G1, increases in the S and G2 phases, maximal levels in the M phase, and a rapid dissipation in the late stage of cell division. Ki-67 has a short half-life and is the most reliable indicator of tumor cell proliferation activity, with its increased expression being associated with increases in tumor malignancy.^[50]^ In this way, the stronger the cell proliferation, the higher the malignancy. In our study, Ki-67 was divided into 2 groups using 15% as the cutoff value. We found that LPP expression in the Ki-67 >15% group was stronger than that in Ki-67 <15% group. Such results suggest that high expression levels of LPP may indicate increased degrees of PD malignancy.

In some diseases, the severity and prognosis may vary as a function of age. For example, there is clear evidence that breast cancer in young women is more aggressive and possesses potentially unique, invasive, and complex biological characteristics.^[51–53]^ And findings from a recent study suggest that youth represents an independent prognostic factor for survival in the diagnosis of breast cancer.^[54]^ Here, we found that the expression intensity of LPP in the middle-aged group was greater than that in the senior group. This result suggests that MPD may be more aggressive in young versus senior patients. While this age difference requires further investigation, we propose 2 possible explanations. As the diagnosis in young female patients is usually delayed, this may lead to belated treatments and poor prognosis, or these may be due to age-related differences in the invasiveness or biological aspects of the disease itself.

In addition to PCs, the typical pathological manifestations of PD include hyperkeratosis and/or hypokeratosis, acanthosis, inflammatory cell infiltration in dermis, and loosening of the spinal layer. We found that the thickness of spinous process and length of epidermal processes in the EMPD group were greater, while the number of PCs per unit area less, than that in the MPD group. However, there were no statistically significant correlations between these indices and LPP expression in the MPD and EMPD groups. Therefore, further research will be required to determine whether LPP is related to these pathological manifestations in PD.

In conclusion, here we show that LPP was mainly expressed in the cytoplasm of PCs with the color of brown and yellow, and the intensity of its expression was significantly greater in MPD versus EMPD samples. This differential expression of LPP between MPD and EMPD may provide a marker to distinguish between these 2 conditions. When combined with results from previous studies examining the role of LPP in breast cancer,^[20]^ we believe that our results provide strong evidence indicating that MPD originates from internal breast cancer. The association of LPP with metastasis and infiltration in many types of cancer suggests that it may also serve as marker to evaluate the severity of the disease, high expression levels of LPP often indicating a high degree of tumor malignancy. Age may represent an independent factor in MPD, with younger MPD patients showing a more aggressive variant of this condition.

Our study has certain limitations. First, the study was conducted at one center and will require data from other regions to corroborate our findings. Second, our results are based on a retrospective analysis, which may lead to selection bias due to various treatment strategies. Therefore, prospective studies will be needed to validate our findings. Third, the limited sample size of our study may have precluded the demonstration of statistically significant differences for some of these variables. Finally, functional studies will ultimately need to be performed to reveal the biological mechanisms of LPP in PD.

## Author contributions

**Data curation:** Ye Liu.

**Formal analysis:** Yangbin Wang.

**Investigation:** Ruiqun Qi.

**Software:** Ruiqun Qi.

**Writing – original draft:** Xiaoyun Mao.

**Writing – review & editing:** Feng Jin.
